# Diet-Induced Rat Model of Gradual Development of Non-Alcoholic Fatty Liver Disease (NAFLD) with Lipopolysaccharides (LPS) Secretion

**DOI:** 10.3390/diagnostics9040205

**Published:** 2019-11-27

**Authors:** Dominika Maciejewska, Agnieszka Łukomska, Karolina Dec, Karolina Skonieczna-Żydecka, Izabela Gutowska, Marta Skórka-Majewicz, Daniel Styburski, Kamila Misiakiewicz-Has, Anna Pilutin, Joanna Palma, Katarzyna Sieletycka, Wojciech Marlicz, Ewa Stachowska

**Affiliations:** 1 Department of Human Nutrition and Metabolomics, Pomeranian Medical University in Szczecin, 70-204 Szczecin, Poland; karolina_dec@wp.pl (K.D.); karzyd@pum.edu.pl (K.S.-Ż.); marta_skorka@o2.pl (M.S.-M.); daniel.styburski@interia.pl (D.S.); palma.01.01@gmail.com (J.P.); ewastachowska.pum@gmail.com (E.S.); 2 Laboratory of Neuroplasticity Nencki Institute of Experimental Biology PAS, 02-093 Warsaw, Poland; agnieszka_lukomska@wp.pl; 3 Department of Medical Chemistry, Pomeranian Medical University in Szczecin, 70-204 Szczecin, Poland; izagut@poczta.onet.pl; 4 Department of Histology and Embryology, Pomeranian Medical University in Szczecin, 70-204 Szczecin, Poland; kamila.misiakiewicz@pum.edu.pl (K.M.-H.); anna.pilutin@pum.edu.pl (A.P.); 5 Faculty of Exact and Natural Sciences, Institute of Biology, University of Szczecin, 70-204 Szczecin, Poland; kasia.sielatycka@gmail.com; 6 Department of Gastroenterology, Pomeranian Medical University, 71-252 Szczecin, Poland; marlicz@hotmail.com

**Keywords:** NAFLD animal model, NAFLD rat model, NASH model, NAFLD model

## Abstract

Background: Non-alcoholic fatty liver disease (NAFLD) is one of the most common liver disorders in industrialized Western countries. The prevalence of the disease is estimated to range from 4% to 46% worldwide. The aim of study was to develop an animal model with gradual NAFLD development. Methods: Sprague-Dawley rats were fed a high-fat and high-cholesterol (HFHCh) diet. The rats from the study and control groups were sacrificed after 2, 4, 8, 12, 16, and 20 weeks of dietary exposure. Results: Analysis of biochemical parameters showed that after only two weeks, ALT and cholesterol concentration in serum were elevated. After 4 weeks, TNF-α and HOMA-IR were significantly higher compared to the control group. NAFLD progression started after 12 weeks of diet-weight gain and increased LPS secretions were noticed. During the experiment, rats induced steatosis (from stage 0/1 after 4 weeks to stage 2/3 after 20 weeks), inflammation (from stage 0/1 after 4 weeks to stage 1/2 after 20 weeks), and fibrosis (from stage 1 after 12 weeks to stage 2 after 20 weeks). Conclusion: We can assume that the presented model based on the HFHCh diet induced gradual development of NAFLD. We confirmed that the animal NAFLD model increases LPS secretions during disease progression.

## 1. Introduction

Non-alcoholic fatty liver disease (NAFLD) is one the most common liver disorders in industrialized Western countries. The prevalence of the disease is estimated to range from 4% to 46% worldwide [1,2]. NAFLD comprises a range of disorders, including simple steatosis without damage of hepatocytes, as well as development of fatty liver with ongoing inflammation and fibrosis. The pathogenesis of NAFLD is a complex process involving numerous factors, such as dyslipidemia [3], insulin resistance, overweight, obesity, mitochondrial dysfunction, oxidative stress, the development of an inflammatory state, the disorders of the metabolism of fat tissue, as well as dysbiosis and genetic factors [4,5]. As many factors contribute to the mechanism of the disease, the “multiple hit” hypothesis is the new and generally accepted theory of NAFLD development [6]. The prevalence of NAFLD in Europe ranges between 20% and 33% among the adult population [7]. The progression of NAFLD is associated with constant exposure to pathogenic factors, endocrine disorders [8], lipid accumulation in the liver, and oxidative stress, which lead to inflammation and hepatocyte damage. Simple steatosis under the inflammatory condition progresses to non-alcoholic steatohepatitis (NASH) [9] and contributes to insulin resistance caused by peripheral tissue lipolysis, triacylglycerols (TG) synthesis, and consequently, increased hepatic uptake of free fatty acids. Further, the fat accumulated in hepatocytes undergoes a peroxidation process, which results in the production of proinflammatory cytokines, directly involved in NASH development. The histopathological features are: (i) large-scale steatosis with the presence of inflammatory changes, (ii) Mallory bodies, (iii) fibrosis and cirrhosis. The severity of these features is correlated with the severity of the disease [9,10]. NASH develops in 5%–7% of the general population and in approximately 7%–30% of NAFLD patients [11]. In comparison to simple steatosis, NASH is an important risk factor for the development of liver cirrhosis and hepatocellular carcinoma (HCC). Within six years of NASH diagnosis, 26%–37% of patients develop symptoms of fibrosis. Every fifth patient with NASH may develop at least partial cirrhosis of the liver, and patients with cirrhosis have a 40%–60% chance of developing HCC. In contrast to NASH patients having 6 times the increased risk of developing HCC, the risk of HCC in individuals diagnosed with simple steatosis does not exceed 0.5% [12].

## 2. Materials and Methods

### 2.1. Animals

The experiment was carried out on 72 male, eight-week-old Sprague-Dawley rats. The rats were randomly assigned to study arms and then separated into plastic cages (3 rats per cage). The rats were kept in 12 h light/darkness cycles in rooms with heating and temperature control, and they had ad libitum access to food and water. The study group (*n* = 36, 6 groups of 6 rats each) received a high-fat and high cholesterol diet (HFHCh) similar to previously described by Xu et al. [13]. In order to maintain the same level of fiber, vitamins, and minerals in both groups (study and control group), the diet consisted of 88 g of standard food (Rodent Lab Chow, PURINA), 10 g of lard oil, 2 g of cholesterol, and additional micro and macro elements. The control group (*n* = 36, 6 groups of 6 rats each) received standard food for laboratory rats (Rodent Lab Chow, PURINA). Table 1 shows sources of energy in the HFHCh diet and control diet.

The rats from the study and control groups were sacrificed after 2, 4, 8, 12, 16, and 20 weeks of dietary exposure. At each time point, 12 animals were sacrificed—6 from the control group and 6 from the study group. The animals were sacrificed by injection of ketamine intraperitoneally. Subsequently, 4 mL of blood was collected to a vial with a gel tube with a clotting accelerator. After 20 min, blood was centrifuged for 10 min in 4 °C, 1200*g*. Serum was quickly collected and frozen in −80 °C. Procedures involving animals were carried out in strict accordance with international standards of animal care guidelines and every effort was made to minimize suffering and the number of animals used. Experiments were approved by the Local Ethical Committee on Animal Testing in Poznan, Poland (approval No. 76/2016, approved on 16 December 2016). No rat died in either group during the whole experimental period.

### 2.2. Histological Evaluation

For histological examination, the livers were immediately taken, fixed in 4% buffered formalin solution, embedded in paraffin, and cut into 4 µm sections. For the morphological analysis (Leica DM5000B, Germany), serial sections of livers were stained with hematoxylin-eosin (HE).

Hepatic fibrosis was assessed by Mallory trichrome methods (Bio-Optica, Italy). Ten light microscopic fields were viewed on each section and scored for the severity of hepatic steatosis and fibrosis [14]. For hepatic steatosis, the following criteria [14] were used: grade 0—no fat; grade 1—steatosis occupying less than 33% of the hepatic parenchyma; grade 2—steatosis occupying less than 34%–66% of the hepatic parenchyma; grade 3—more than 66% of the hepatic parenchyma. The following criteria were used to evaluate the staging of hepatic fibrosis: 0—none; 1—mild, zone 3, perisinusoidal; 2—moderate, zone 3, perisinusoidal; 3—portal/periportal; 4—bridging fibrosis [14]. For inflammatory cell infiltration, the following criteria were used: grade 0—none; 1—1/2 foci/field; 2—3/4 foci/field; 3—more than 4 foci/field [13].

### 2.3. Biochemical Measurements

All biochemical parameters were measured using an enzyme-linked immunosorbent assay kit (ELISA). An Alanine Transaminase Colorimetric Activity Assay Kit, Cholesterol Fluorometric Assay Kit, and Glucose Colorimetric Assay Kit were purchased from Cayman Chemical, a TNF-alpha Quantikine ELISA Kit and an Insulin ELISA Kit from R&D Systems, and a Rat Lipopolysaccharides (LPS) ELISA Kit from MyBioSource. Insulin resistance (HOMA-IR) was calculated according to the following formula: fasting serum glucose × fasting serum insulin/22.5.

### 2.4. Statistical Analysis

Various statistical methods were applied according to the type of experiments performed as briefly described below.

ELISA analysis: The statistical analysis was performed using Statistica 12.0 software. The Shapiro–Wilk test was used to inspect the normal distribution. Since the distribution did not deviate from the norm, parametric tests were used. The results are presented as mean values and standard deviation (SD). In order to check the differences between the studied parameters, the Student *t* test (*t*.test) was used both for the paired and unpaired data. In order to estimate the correlation, the Pearson’s correlation test was used. The values of *p* < 0.05 were considered as statistically important. In reference to the results which were not statistically significant, the abbreviation NS (not significant) was used.

Histological statistics: For comparison of categorical variables, the chi-squared test was used. The difference between the two proportions and a 95% confidence interval (CI) for this difference; the CI was calculated according to the recommended method given by [15]. Chi-squared test and P value—when this P value is less than 0.05, the conclusion was that the two proportions differ significantly.

## 3. Results

### Hepatic Steatosis, Inflammation, and Fibrosis

**Figure 1:**
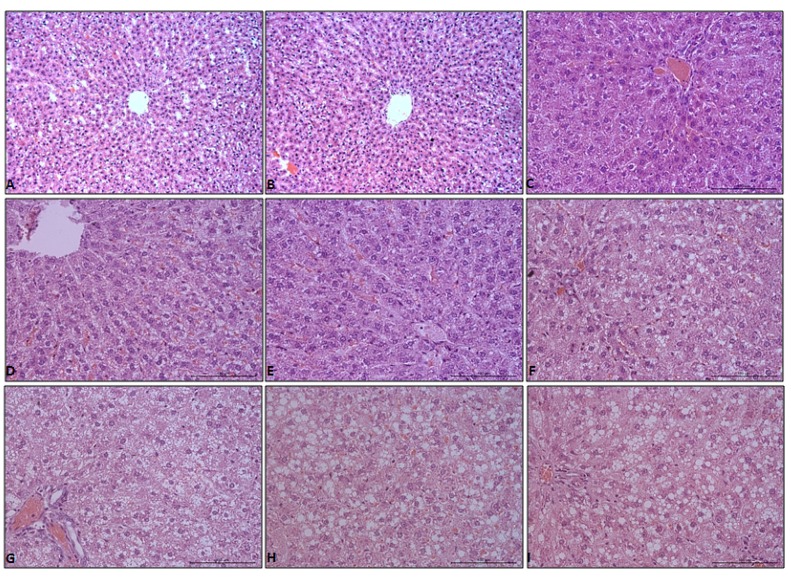
Hematoxylin-eosin (HE) stain of liver tissue from control (**A**–**C**), HFD livers at weeks 2 (**D**), 4 (**E**), 8 (**F**), 12 (**G**), 16 (**H**), and 20 (**I**). Objective magnification: **A**,**B** ×20; **C**–**I** ×40.

There were no abnormalities in the liver morphology of the control group. Hepatocytes were arranged in typical plates of cords that branch and anastomose in a continuous labyrinth. The lobules resemble a hexagon with portal tracts at the corners and a centrally located central vein, as shown in Figure 1A–C.

There were no stages of hepatic steatosis, as shown in Figure 1D and Table 2, and inflammation, as shown in Table 3, at 2 weeks in livers of the high fat diet (HFD) group rats. Starting from 4 weeks, all of the HFD group rats showed an increase in lipid accumulation and pronounced steatosis in the liver, which was characterized by prominent lipid droplet deposition in cytoplasm, as shown in Figure 1E–I. All differences in livers of HFD groups were statistically significant for the control groups, as shown in Table 2. At this time point, mild hepatic lobular inflammation (grade 1) was present, as shown in Table 3.

Livers of HFD rats at 4 weeks showed mild changes. At this time point, only 25% of the evaluated histological fields developed stage 1 hepatic steatosis, as shown in Table 2. After 8 weeks of the experiment, the appearance of changes corresponding to grade 2 hepatic steatosis was observed in HFD rats, as shown in Table 2, as well as more severe hepatic lobular inflammation being present, as shown in Table 3. From week 12 to 20 of the HFD group rats, grades 2 and 3 hepatic steatosis were present in all evaluated histological fields. Particularly strongly developed stage 3 hepatic steatosis was visible in the 20 HFD group, as shown in Figure 1I and Table 1. Hepatic lobular inflammation after 12 weeks of the experiment was more severe than those in 4 HFD and 8 HFD, and appearance of changes corresponding to grade 2 was observed. Also, lobular, as well as portal, inflammation was observed in livers of HFD rats at 16 and 20 weeks, with changes corresponding still to grade 2, as shown in Table 3.

**Figure 2:**
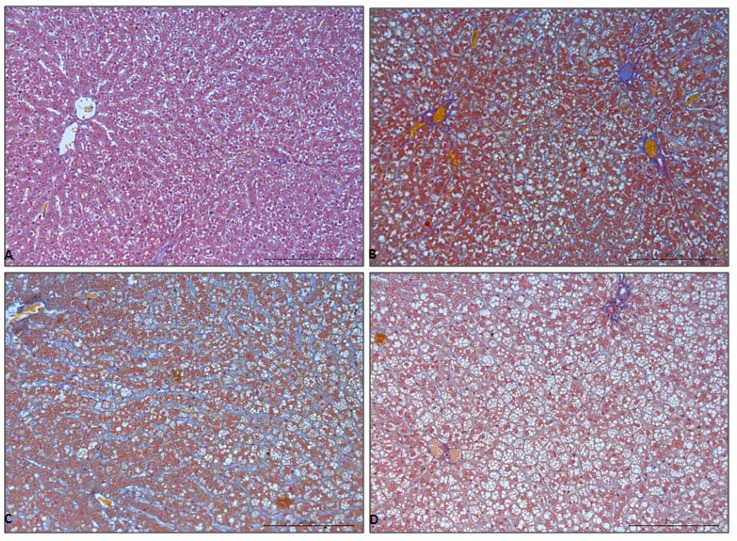
Mallory trichrome stain of liver tissue from control (**A**) and HFD livers at weeks 12 (**B**), 16 (**C**), 20 (**D**). Objective magnification ×20.

There were no histological signs of hepatic fibrosis in the liver of control group, as shown in Figure 2A and Table 2. Hepatic fibrosis was also not observed at week 2, 4, and 8 in the HFD group of rats, as shown in Table 2. After 12 weeks of HFD feeding, mild and moderate, perisinusoidal fibrosis was present, as shown in Figure 2B–D and Table 2. There were no histological signs of portal/periportal and bridging fibrosis representing stages 3 and 4 hepatic fibrosis, respectively, as shown in Table 2.

Changes of biochemical parameters during the experiment are shown in Table 4.

## 4. Discussion

### 4.1. Selection of an Appropriate High Fat Dietary Model of NAFLD

A high fat (HFD) diet, which includes 30%–75% of total calories derived from saturated fat, induced metabolic changes leading to fat storage, insulin resistance, and NAFLD development. Feeding time duration and combination of fatty acids in the diet are important factors that determine the intensity of glucose intolerance, dyslipidemia, proinflammatory cytokine secretion, and fat accumulation [16]. However, the HFD dietary regime may give variable results, which depend on rodent species and strain. Sprague-Dawley rats fed a HFD develop NAFLD and NASH symptoms, which is associated with their diet dependent susceptibility to obesity [17].

However, a long lasting high saturated fat diet did not induce hepatic steatosis and NASH in Wistar rats [18]. The HFD with a cholesterol addition (15% fat, 1% cholesterol) leads to increased hepatic fat deposition with little inflammation and no fibrosis [19]. Kitamori et al. developed a HFHCh diet rat model of NASH in which 2 weeks of dietary intervention induced fat accumulation and lobular inflammation, while 14 weeks of the diet was associated with ballooning degeneration and severe fibrosis [20]. The data are mounting that cholesterol is an important factor of NASH development. Moreover, direct lipotoxicity of free fatty acids and other lipids increase the inflammation process, as well as cholesterol synthesis in the liver. Therefore, the dietary NAFLD model, associated with NASH development, contains cholesterol as one of the “hit” factors in the hypothesis of multiple-hits in NAFLD progression [21].

Our dietary pattern of HFD contained 50% of energy from carbohydrates, 20% from protein, and 30% from fat. Also, 30% of fat energy was mainly lard oil and cholesterol (10 g of lard oil + 2 g of cholesterol with 88 g of standard food). Of note, similar HFHCh diets were described by Xu et al., where 52% of energy came from carbohydrates, 18% from protein, and 30% from fat. This diet also contained 10 g of lard and 2 g of cholesterol per 88 g of standard food [13]. In our study, the time of dietary exposure lasted 4, 8, 12, 16, 24, and 48 weeks. During the study stage, NAFLD ranged from simple steatosis to NASH and advanced fibrosis [13], which can be observed our study. However, in order to avoid deficiencies associated with a 12% reduction of minerals and vitamins (because of the use of only 88 g of standard food), the proper levels of nutrients were added to our HFHCh intervention. Moreover, we established the fiber level as 3.5% for both the HFHCh and control group. It is important to report that the proper level of fiber is essential for maintaining the good condition of the gut and all of the digestive system [22]. The microbiome alteration, which is caused by insufficient amount of fiber, is one of the important factors of NAFLD progression [23,24].

### 4.2. Exposure for the HFHCh Diet

The 2 week diet did not cause steatosis, however, in relation to the control group, animals from the study group showed increased levels of ALT and serum cholesterol. After the 4 week intervention, besides increased concentration of cholesterol and ALT, we observed an increase in insulin resistance (HOMA-IR, 1.3 ± 0.4 vs. 0.95 ± 0.31, HFHCh group vs. control) and increased TNF-α concentration. Increase of these parameters were also noticed after 8 weeks of dietary intervention.

**Figure 3:**
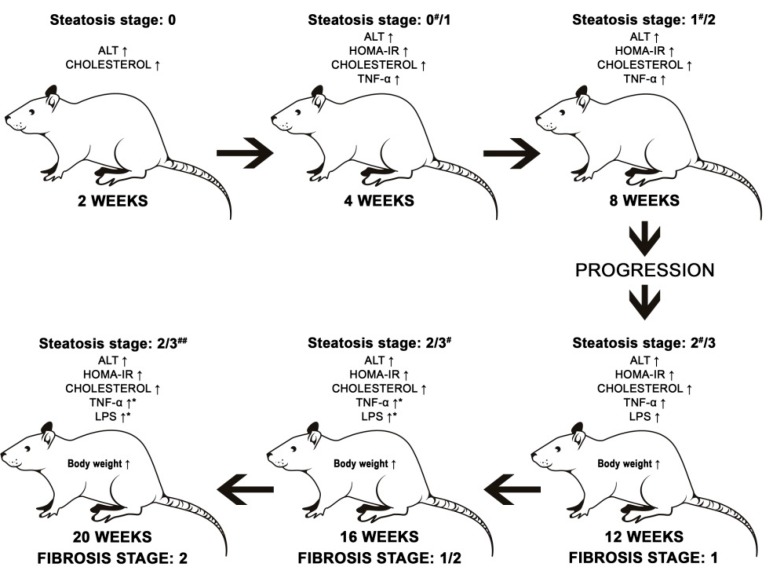
Progression of liver steatosis.

We did not see statistically significant differences between 2, 4, and 8 weeks in the rest of the measured parameters. However, we noticed that there was a progression of liver steatosis in the following pattern: 2 weeks—0% of steatosis, 4 weeks—25% of evaluated histological fields developed stage one of hepatic steatosis, 8 weeks—78.3% of evaluated histological fields developed stage one and 21.7% developed stage two. After 8 weeks, 20% of evaluated histological fields developed a first stage of inflammation score. In the order to evaluate the mechanism of NAFLD, associated with simply steatosis without fibrosis and massive inflammation, 8 weeks of the HFHCh dietary approach was sufficient. The schematic changes of measured parameters along with dietary invention are shown in Figure 3.

After 12 weeks of exposure to a HFHCh diet, rats revealed body weight gain, which was significantly higher in comparison to the 2, 4, and 8 week groups. The body mass after 16 and 20 weeks was significantly increased compared to the control groups, but we did not see significant progression between 12 and 20 weeks. The 12 week diet increased also lipopolysaccharides (LPS) concentration in serum. LPS are complex amphiphilic molecules released from bacterial cell walls by shedding or through bacterial lysis [25]. Normally, LPS do not penetrate across the healthy intestinal epithelium, except for the intestinal permeability disorders. Of importance, the defective gut barrier allows paracellular flux of LPS and other luminal antigens [26], and increased levels of LPS in serum plays an important role in NAFLD progression. Fukunishi et al. studied the role of LPS in NAFLD development. Increased levels of LPS caused a decrease in adiponectin concentration, an increase in leptin concentration, and greater expression of fatty acid synthase and transcription factors of de novo lipogenesis in the liver [27,28]. After 16 and 20 weeks of experiment, the significant progression of cholesterol concentration, TNF-α concentration, and LPS secretion were observed. Histological evaluation showed that the 12 week intervention induced steatosis in all rats studied. Also, 63.33% of evaluated histological fields developed the second, and 36.67%, the third stage of steatosis. This tendency persisted until the end of the experiment, and after 20 weeks, 75% of evaluated histological fields developed the third stage of steatosis. After 16 weeks, more than 50% of histological fields revealed the first and second stages of inflammation scores, and 80% manifested fibrosis, as shown in Figure 3.

## 5. Conclusions

We can assume that the presented model based on a HFHCh diet induced gradual development of NAFLD. During NAFLD progression, rats induced steatosis, inflammation, and fibrosis. For the first time in animal NAFLD models, we confirmed increased LPS secretion during NAFLD progression.

## Figures and Tables

**Table 1:** Main components in the high-fat and high cholesterol (HFHCh) diet and control group.

Component	HFHCh	Control
Carbohydrates [%E]	50	65
Fat [%E]	30	10
Protein [%E]	20	25
Fiber [%]	3.5	3.5
Cholesterol [g/100g]	2	0
Methionine [g/100g]	0.65	0.65
Choline [g/100g]	0.2	0.2

**Table 2:** Scores of hepatic steatosis and hepatic fibrosis staging.

Group	Week	N (*n*)	Histological Grades of Steatosis Number of Evaluated Histological Fields (Percentage of Grade of Steatosis)				Fibrosis Stage (N)				
			0	1	2	3	0	1	2	3	4
Control	2–20	36 (360)	360 (100)	0 (0)	0 (0)	0 (0)	36	0	0	0	0
HFHCh	2	6 (60)	360 (100)	0 (0)	0 (0)	0 (0)	6	0	0	0	0
	4	6 (60)	45 (75) *^,a^	15 (25) *^,a^	0 (0)	0 (0)	6	0	0	0	0
	8	6 (60)	0 (0) *^,a,b^	47 (78.3) *^,a,b^	13 (21.7) *^,a,b^	0 (0)	6	0	0	0	0
	12	6 (60)	0 (0) *^,a,b^	0 (0) ^b,c^	38 (63.33) *^,a,b,c^	22 (36.67) *^,a,b,c^	1	4	1	0	0
	16	6 (60)	0 (0) *^,a,b^	0 (0) ^b,c^	26 (43.33) *^,a,b,c,d^	34 (56.67) *^,a,b,c,d^	1	3	2	0	0
	20	6 (60)	0 (0) *^,a,b^	0 (0) ^b,c^	15 (25) *^,b,d,e^	45 (75) *^,a,b,c,d,e^	1	1	4	0	0

Data of steatosis are expressed as counts and percentages in parentheses. N—number of animals, (*n*)—number of evaluated histological fields (10 fields per animal). * *p* < 0.0001 vs. control, ^a^ *p* < 0.0001 vs. 2 HFHCh, ^b^ *p* < 0.0001 vs. 4 HFHCh, ^c^ *p* < 0.05 vs. 8 HFHCh, ^d^ *p <* 0.05 vs. 12 HFHCh, ^e^ *p <* 0.05 vs. 16 HFHCh.

**Table 3:** Data of inflammation are expressed as counts and percentages in parentheses.

Group	Week	N (*n*)	Inflammation ScoreNumber of Evaluated Histological Fields (Percentage of Grade of Inflammation)			
			0	1	2	3
Control	2–20	36 (360)	360 (100)	0 (0)	0 (0)	0 (0)
HFD	2	6 (60)	360 (100)	0 (0)	0 (0)	0 (0)
	4	6 (60)	48 (80) **	12 (20) **	0 (0)	0 (0)
	8	6 (60)	41 (68.33) ***^,a^	18 (30) ***^,a^	1 (1,67)	0 (0)
	12	6 (60)	38 (63.33) ***^,a,f^	18 (30) ***^,a^	4 (6.67) *^,c,f^	0 (0)
	16	6 (60)	28 (46.66) ***^,a,d,h^	25 (41.67) ***^,a,f^	7 (11.67) **^,b,e,h^	0 (0)
	20	6 (60)	26 (43.34) ***^,a,d,g,i^	26 (43.33) ***^,a,e^	8 (11.33) **^,b,e,h^	0 (0)

N—number of animals (n)—number of evaluated histological fields (10 fields per animal). HFD— high fat diet. *** *p <* 0.0001 vs. control, ** *p <* 0.001 vs. control, * *p <* 0.01 vs. control, ^a^ *p <* 0.0001 vs. 2 HFHChD, ^b^ *p <* 0.001 vs. 2 HFHChD, ^c^ *p <* 0.05 vs. 2 HFHChD, ^d^ *p <* 0.0001 vs. 4 HFD, ^e^ *p <* 0.001 vs. 4 HFHChD, ^f^ *p <* 0.05 vs. 4 HFHChD, ^g^ *p <* 0.001 vs. 8 HFHChD, ^h^ *p <* 0.05 vs. 8 HFHChD, ^i^ *p <* 0.05 vs. 12 HFHChD.

**Table 4:** Biochemical parameters.

**Time Points**	**Weight [g]**		**ALT [U/L]**		**HOMA-IR**	
	**CONTROL**	**HFHCh**	**CONTROL**	**HFHCh**	**CONTROL**	**HFHCh**
**2 weeks**	401.66 ± 10.57	400 ± 14.14	18.8 * ± 0.06	26.6 * ± 0.73	0.69 ± 0.25	1.02 ^5,6^ ± 0.46
**4 weeks**	380.83 ± 17.89	424.16 ± 32.07	19.4 * ± 0.09	24.8 * ± 0.18	0.95 * ± 0.31	1.30 * ± 0.41
**8 weeks**	408.33 ± 10.67	420.83 ± 16.69	18.2 * ± 0.04	23.6 * ± 0.26	0.48 * ± 0.10	1.48 * ± 0.53
**12 weeks**	420.00 * ± 12.90	463.33 *^1,2,3^ ± 11.05	19.0 * ± 0.05	22.7 * ± 0.29	0.73 * ± 0.215	1.30 * ± 0.70
**16 weeks**	418.33 * ± 13.44	477.50 *^1,2,3^ ± 19.09	18.4 * ± 0.07	30.4 * ± 0.57	0.66 * ± 0.32	1.71 * ± 0.48
**20 weeks**	420.00 * ± 16.33	496.67 *^1,2,3^ ± 17.95	18.3 * ± 0.92	27.9 * ± 0.49	0.69 * ± 0.38	2.07 * ± 0.72
**Time Points**	**CHOLESTEROL [mg/dL]**		**TNF-α [pg/mL]**		**LPS [ng/mL]**	
	**CONTROL**	**HFHCh**	**CONTROL**	**HFHCh**	**CONTROL**	**HFHCh**
**2 weeks**	60.47 * ± 17.65	101.24 *^,5,6^ ± 19.77	122.34 ± 9.49	149.51 ^5,6^ ± 17.62	1.47 ± 1.88	2.62 ^5,6^ ± 2.59
**4 weeks**	64.77 * ± 6.07	111.31 *^,5,6^ ± 14.66	118.6 * ± 6.19	142.53 ^5,6^ * ± 20.57	2.17 ± 1.61	2.57 ^5,6^ ± 2.00
**8 weeks**	46.13 * ± 22.79	112.38 *^,5,6^ ± 16.54	124.30 * ± 7.12	136.74 *^,5,6^ ± 10.41	1.48 ± 1.84	2.71 ^5,6^ ± 0.98
**12 weeks**	49.10 * ± 12.41	121.52 *^,5,6^ ± 19.77	125.41 * ± 13.26	141.11 *^,5,6^ ±16.51	2.03 * ± 2.84	3.37 *^6^ ± 3.32
**16 weeks**	68.48 * ± 15.60	185.79 *^,1,2,3,4^ ± 29.92	124.14 * ± 17.40	165.34 *^,1,2,3,4,6^ ± 16.12	1.77 * ± 1.47	3.97 *^,1,2,3,6^ ± 2.17
**20 weeks**	72.63 * ± 11.66	203.16 *^,1,2,3,4^ ± 64.42	129.13 * ± 18.12	241.58 *^,1,2,3,4,5^ ± 98.49	2.33 * ± 1.91	8.82 *^,1,2,3,4,5,6^ ± 3.25

* *p* < 0.05 between the control and non-alcoholic fatty liver disease (NAFLD) group, *p* < 0.05 between particular subgroup: ^1^—2 weeks, ^2^—4 weeks, ^3^—8 weeks, ^4^—12 weeks, ^5^—16 weeks, ^6^—20 weeks. LPS— lipopolysaccharides.
